# Influence of Axial Depth of Cut and Tool Position on Surface Quality and Chatter Appearance in Locally Supported Thin Floor Milling

**DOI:** 10.3390/ma15030731

**Published:** 2022-01-19

**Authors:** Mikel Casuso, Antonio Rubio-Mateos, Fernando Veiga, Aitzol Lamikiz

**Affiliations:** 1 TECNALIA, Basque Research and Technology Alliance (BRTA), Parque Científico y Tecnológico de Gipuzkoa, E20009 Donostia-San Sebastián, Spain; antonio.rubio@tecnalia.com (A.R.-M.); fernando.veiga@tecnalia.com (F.V.); 2 Department of Mechanical Engineering, University of the Basque Country (UPV/EHU), E48013 Bilbao, Spain; aitzol.lamikiz@ehu.eus

**Keywords:** finish milling, chatter, part quality, AA2024 floor milling

## Abstract

Thin floor machining is a challenging and demanding issue, due to vibrations that create poor surface quality. Several technologies have been developed to overcome this problem. Ad hoc fixtures for a given part geometry lead to meeting quality tolerances, but since they lack flexibility, they are expensive and not suitable for low manufacturing batches. On the contrary, flexible fixtures consisting of vacuum cups adaptable to a diversity of part geometries may not totally avoid vibrations, which greatly limits its use. The present study analyses the feasibility of thin floor milling in terms of vibration and roughness, in the cases where milling is conducted without back support, a usual situation when flexible fixtures are employed, so as to define the conditions for a stable milling in them and thus avoid the use of ad hoc fixtures. For that purpose, the change of modal parameters due to material removal and its influence on chatter appearance have been studied, by means of stability lobe diagrams and Fourier Transform analysis. Additionally, the relationship between surface roughness and chatter frequency, tooth passing frequency, and spindle frequency have been studied. Ploughing effect has also been observed during milling, and the factors that lead to the appearance of this undesirable effect have been analyzed, in order to avoid it. It has been proven that finish milling of thin floors without support in the axial direction of the mill can meet aeronautic tolerances and requirements, providing that proper cutting conditions and machining zones are selected.

## 1. Introduction

Achieving good surface quality in final parts is a widespread concern among manufacturers of all kinds. The milling of thin parts is an especially critical issue [1], since the stiffness of these parts is low, which eases the appearance of vibration, as chatter and forced vibration, which negatively affect final surface quality and tool life [2]. This problem can lead to the rejection of these parts or to the necessity of reprocessing, which entails high costs in terms of material, time, and energy.

However, as Kolluru and Axinte [3] and Irene Del Sol et al. [4] have pointed out, chatter avoidance in thin parts has traditionally focused on thin wall milling, whereas thin floor milling has been relegated, even though its importance is high in fields such as aeronautic and aerospace industries, where pockets are milled in parts as aircraft structures in order to lighten them and must comply with stringent surface requirements. Precisely, mechanical milling appears as an alternative to chemical milling for thin floors, which is hazardous and pollutant [5,6].

Chatter is a self-excited vibration that appears due to the dynamic excitation produced as a consequence of the chip thickness variation caused by the periodic irregular surface generated by the precedent tool tooth pass [7]. Mainly, three types of solutions have been proposed to cope with vibrations in mechanical milling of thin floors: fixture design aiming to increase the stiffness of the part, damping systems aiming to dissipate vibration energy, and the prediction of stable machining conditions [8]. Both fixture and damping systems can be ad hoc for a given geometry or flexible and adjustable for different ones. On the one hand, if they are ad hoc, they are able to avoid vibrations as they completely support thin floors, but they also require investments that are neither feasible nor profitable when manufacturing low batches, as it is usual in aeronautic and aerospace sectors [9]. On the other hand, flexible fixtures usually consist of actuators that support the part by means of vacuum cups. In such a layout, due to the distance between actuators, some zones of the part may be without back support. Chatter vibration can appear easily in this situation of extremely low stiffness. Due to this fact, this kind of fixture is usually discarded for high precision milling, as it is usual in aeronautic and aerospace sectors, and they are employed only for other operations, such as drilling, trimming, or for milling processes that do not require demanding tolerances [10]. As a consequence, the potential of flexible fixtures has not been fully harnessed yet. A deeper analysis consisting of predicting and selecting proper cutting conditions and areas leading to a stable and precise machining can be a solution [2]. It would make feasible thin floor milling even in the situation of such a low stiffness that happens in flexible fixtures.

In this line, F.J. Campa et al. [11] suggest the mathematical analysis of the cutting process for thin floors, by means of stability lobes diagram (SLD) determination, which allows the selection of the pair of values of spindle speed and axial depth of cut leading to the most stable and productive machining. In the case of parts with low stiffness, as thin floors are, the dynamic parameters of the part continuously vary during milling, since both mass and stiffness decrease. So, in addition to the depth of cut and spindle speed, Bravo et al. [12] suggest taking into account the geometric state of the part during milling, thus leading to three-dimensional SLD. This approach is followed in the present study, due to the low stiffness and the relatively high material removal rate that thin floors undergo.

The experimental setup has been planned to test different cutting conditions in a context of extremely low stiffness, which is a thin floor simply screwed in its corners and without back support. It emulates a thin floor supported by four clamping vacuum cups, as it happens in flexible fixtures. The objective is to analyze the milling of thin floors aiming to optimize the surface quality achieved and to avoid chatter vibration. For that purpose, thin plates have been pocket milled. Vibration and surface roughness results have been measured, discussed, and compared to the available bibliography. Finally, some guidelines are proposed to make such thin floor milling feasible.

## 2. Materials and Methods

### 2.1. Tested Parts

Aiming to emulate a real industrial case, a series of pocketing tests were carried out in metal thin plates. Each thin plate is a square metal sample of 85 × 85 mm^2^ and 2 mm thick, very similar to the samples used by Del Sol et al. [13] and Rubio-Mateos et al. [14]. The material is aluminum alloy UNS 2024-T3, widely employed in the aeronautic industry.

**Figure 1:**
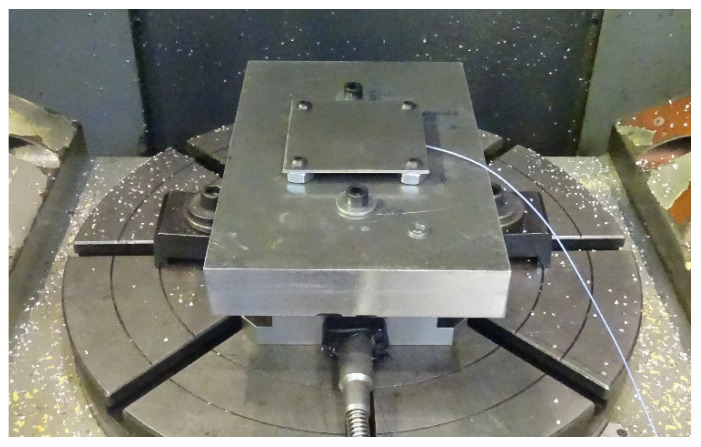
General setup of a sample to be machined, with the accelerometer below.

Prior to any machining operation, each thin floor was drilled near its corners to make holes of 7 mm in order to screw it to an intermediate rigid block that maintains the stability of the process. Each thin floor was elevated, so the screws were its only support and clamping during the entire machining process, which causes a reduction of stiffness in the axial direction of the mill. The centers of the screws form a square of 69 × 69 mm^2^. This layout is an extrapolation of the milling of a thin floor locally supported by four vacuum cups. The general setup is shown in Figure 1.

### 2.2. Methodology

The methodology employed in the present study of the pocket machining of thin plate samples comprises various steps.

First, the frequency response function (FRF) of the tool was measured prior to the machining, as well as the FRFs of the thin plates in four consecutive stages of the machining, in order to calculate the three-dimensional stability lobe diagrams (3D-SLD) of the system. Besides, the vibration of the sample during machining was continuously monitored, and it is used to calculate its Fast Fourier Transform (FFT). To this point, the methodology is similar to the one followed by Kolluru and Axinte [15] to analyze the impact dynamics in the machining of low rigidity workpieces, by means of the determination of dominant modes.

In addition, a roughness analysis was conducted at the end of the present study, in order to correlate it to the vibrations of the samples during machining. The general overview of the tasks is shown in Table 1.

### 2.3. Machining Operation

The machining operation that was performed in each thin floor consisted in a dry milling of a pocket of 50 × 50 mm^2^ by an outward helicoidal strategy, which is one of the most suitable machining strategies regarding final roughness, accuracy, and process time, as Del Sol et al. [16] have proved. This strategy follows the guidelines of Herranz et al. [7] taking advantage of the rigidity of the uncut part, which is higher near the screws.

**Figure 2:**
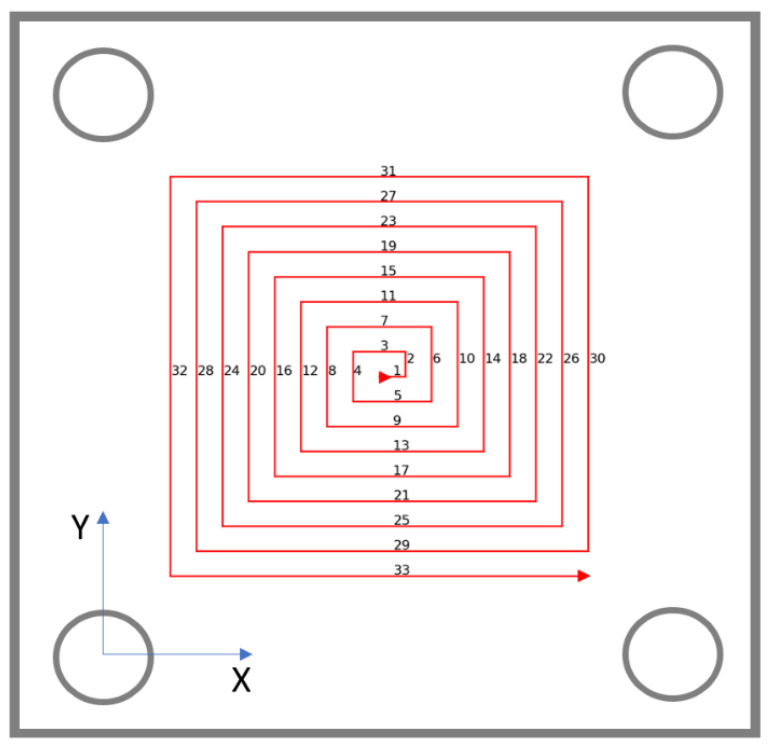
Followed outward helicoidal machining strategy.

The strategy comprised 33 straight cutting passes, consecutively numbered in Figure 2, plus an initial brief drilling in the center in order to start the milling.

The pocket machining was performed in a 5-axis NC center Ibarmia ZV 25U600 EXTREME, with a two flutes bull-nose end-mill Kendu 4400, which has a 10 mm diameter, 30° helix angle and 2.5 mm edge radius (*r*).

Regarding the cutting conditions, conservative ones were selected, as they lead to lower part distortion and lower surface roughness in an aluminum alloy [17,18]. Even though the machining time is higher, this factor is out of the scope of the present study. So, the spindle spins at 4000 rpm and the feed rate was 800 mm/min (0.1 mm/tooth). The radial immersion of the mill was 5 mm.

A different axial depth of cut was employed for pocketing each thin floor (1, 0.8, 0.4 and 0.2 mm), in order to compare the influence of different material removal rates both in vibrations and in achieved final roughness. Due to the depths of cut employed, the thin plates can be named as shown in Table 2. Their remaining features, both in material and in machining operation, are identical.

### 2.4. Vibration Monitoring, FRF Obtention and SLD Calculation

SLDs of the thin plate samples are calculated from their FRFs, which quantify the response of the sample–tool–spindle–machine system to an excitation. Nevertheless, the calculation of the SLDs of parts with low thickness as employed thin plates entails two phenomena. The first one is that the stiffness of the part is lower than the stiffness of the cutting tool, so chatter vibration is mostly affected by the dynamic properties and critical modes of the part [19]. The second phenomenon is the ratio of material removal, which is high compared to the global volume of the part and that leads to a continuous change in its modal parameters and FRF during machining [20]. In order to consider this feature, there are two possibilities. The first one is the use of Finite Element Analysis (FEA) that aims to simulate the FRFs of the samples. This option was used and validated by Dang et al. [21], who consider the second possibility impractical, that is, the Experimental Modal Analysis (EMA), which aims to measure the FRFs of the samples conducting several impact hammer tests during machining and measuring the response of the system with an attached accelerometer [22]. Nevertheless, FEA also entails the requirement of high computational resources and time expended, so EMA is a suitable option, especially for short machining with a simple setup as the one presented in this study. This option was also followed by Qu et al. [23].

Consequently, aiming to obtain the SLDs of the system, several FRF tests were conducted at different stages of the machining. In each test, an impact hammer hit the sample at its top center, while an accelerometer placed at the bottom center registers the corresponding data. The first test was performed prior to any machining operation, the second one was performed after cutting pass 5, the third one after cutting pass 17, and the last one with the pocket completed, so four FRF tests were performed in each sample. As a consequence of performing each test in different stages of the machining, the calculated SLDs take into account not only the axial depth of cut and the spindle speed, but also the position reached by the tool along the cutting path.

The accelerometer is a uniaxial PCB model 352C22 with a measuring range from 1 to 10 kHz and a sensitivity of 1.0 mV/(m/s^2^), located at the bottom center of the thin floor. This accelerometer was also used to continuously monitor vibrations during machining.

The FRF was obtained only in the thick direction or Z direction of the sample, which is considered to be overwhelmingly less rigid than the others, so it is regarded that the workpiece only moves along this direction, an assumption also followed by Seguy et al. [24] and Arnaud et al. [25].

In the case of the tool, the FRF was obtained in its three spatial directions, gluing the accelerometer to the tool. As the FRF of the tool does not vary during machining, a single impact test before machining suffices for obtaining it.

Aiming to determine the machining conditions leading to chatter vibration, stability lobes were calculated. Since modal parameters of the samples vary during material removal, several stability lobes were obtained corresponding to different machining stages, precisely, the machining stages where FRF tests were undertaken, enabling the calculation of SLDs that also take into account the position reached by the tool along the cutting path. This kind of 3D-SLD was also obtained by Campa et al. [11], analyzing thin floor milling.

Thus, these SLDs show the maximum chatter-free axial depth of cut regarding each spindle speed for a given stage of the machining. They have been obtained by applying the three-dimensional mono-frequency model [26,27], and combining the FRF of the samples with the FRF of the tool.

The forces used in this calculation have been obtained employing the mechanistic approach [28,29], which relates the force components (tangential [$F_{t}$], radial [$F_{r}$] and axial [$F_{a}$]) acting in each differential element *j* of the edge of the mill during machining to the feed per tooth ($f_{z}$), and length (dS), angular position (Φ) and axial depth of cut (dz) of the element *j*.

(1) $${\left[\begin{array}{c} dF_{t}\left(\Phi ,z\right)\\ dF_{r}\left(\Phi ,z\right)\\ dF_{a}\left(\Phi ,z\right) \end{array}\right]}_{j}=\left[\begin{array}{c} K_{te}\\ K_{re}\\ K_{ae} \end{array}\right]\cdot \;dS\left(z\right)+\left[\begin{array}{c} K_{tc}\\ K_{tc}\\ K_{tc} \end{array}\right]\cdot \;f_{z}\;\cdot \;\sin\Phi (\Phi_{j},z)\;\cdot \;dz$$

This relation is based on the friction ($K_{te},\;K_{re},\;K_{ae}$) and shearing ($K_{tc},\;K_{rc},\;K_{ac}$) cutting coefficients, which have been obtained prior to the pocketing operation. Precisely, they were obtained solving the equations using force values measured in a previous grooving test, taking a constant value for the spindle speed of 4000 rpm, which is the speed employed in the pocketing tests.

Bull-nose end mills have a lead angle that varies from 0° to 90°. For the case of the mill employed in the present study, these values would correspond to 0 and 2.5 mm of the axial depth of cut, respectively. Altintas [26] suggested that an average value of 45° could be taken. Since in the present study the axial depth of cut varies only from 0.2 mm to 1 mm, a lower constant lead edge angle could be considered. Following Rubio-Mateos et al. [30], a constant lead edge angle of 20° is taken.

### 2.5. Roughness Measurement

**Figure 3:**
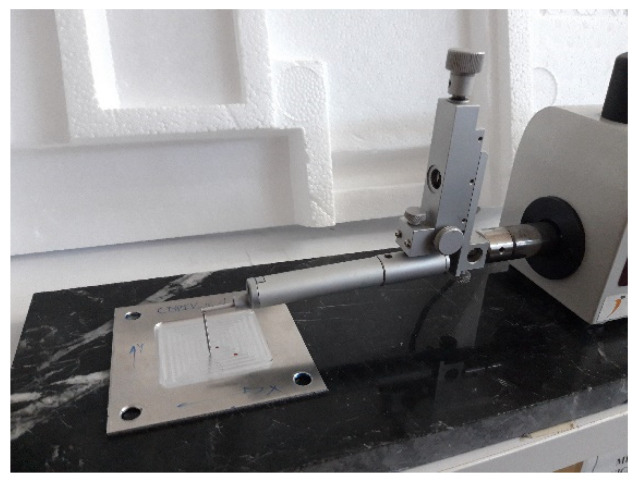
Measurement of final roughness in a sample.

**Figure 4:**
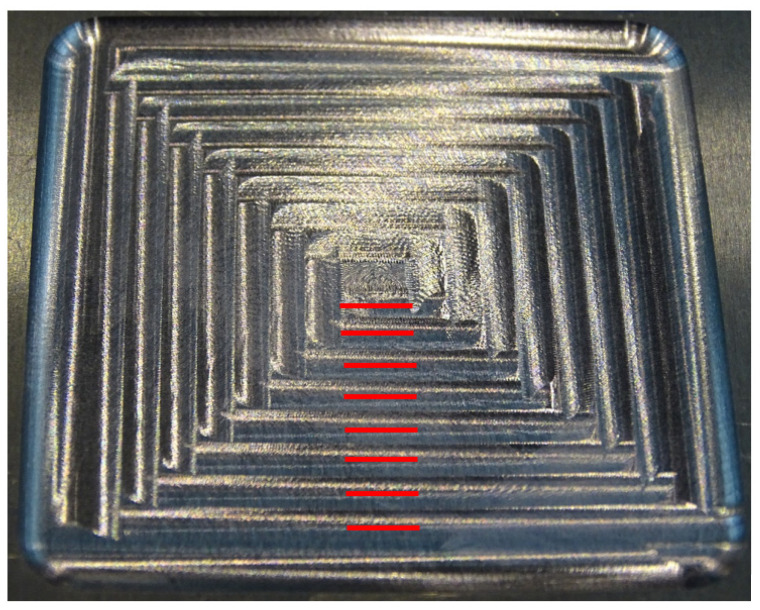
Roughness measurement lines.

The final roughness of the samples after machining was measured in a Mitutoyo Surftest SV-2000 equipment (Figure 3). The roughness profile and the average roughness value (*R_a_*) were measured along 4 mm in the middle of cutting passes 5, 9, 13, 17, 21, 25, 29 and 33. In Figure 4 the accurate lines where roughness was measured can be seen.

### 2.6. Roughness Model

A surface roughness model is proposed to explain its causes and origins, based on the model suggested by Rubio-Mateos et al. [14].

In the experimental setup employed, the roughness in the samples can have three origins. Firstly, the theoretical average roughness (*R_h_*) generated by the tool geometry and the feed per tooth. Secondly, the roughness generated as a consequence of the forced vibration and the axial displacement of the sample (*R_f_*). The axial displacement can happen due to the deflection of the sample or to the relative displacement between sample and fixture. And thirdly, the roughness caused by chatter vibration (*R_c_*). So, the global average roughness (*R_a_*) can be defined as:

(2) $$R_{a}=R_{h}+R_{f}+R_{c}$$

The floor theoretical average roughness is defined as:

(3) $$R_{h}=\frac{f_{z}^{2}}{32r}$$

If chatter-free conditions are guaranteed, roughness caused by chatter is zero, so the difference between measured average roughness and theoretical average roughness will be caused by the axial displacement of the sample. Also, this axial displacement can be considered constant for a given setup and cutting conditions, so the chatter roughness will be the roughness surplus generated at chatter conditions. Cross effects, as well as the axial displacement and the vibration of the tool, are neglected.

## 3. Results and Discussion

### 3.1. SLD Analysis

**Figure 5:**
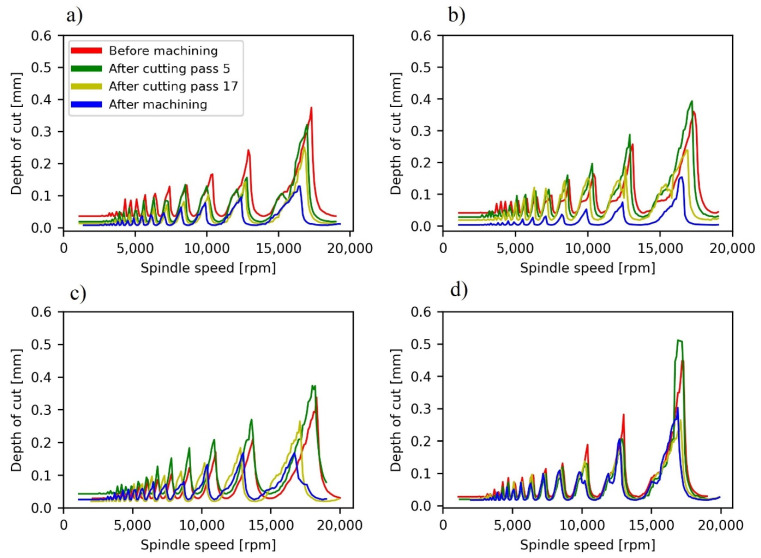
Stability lobe diagrams before, during and after pocket milling. (**a**) TP10. (**b**) TP08. (**c**) TP04. (**d**) TP02.

**Figure 6:**
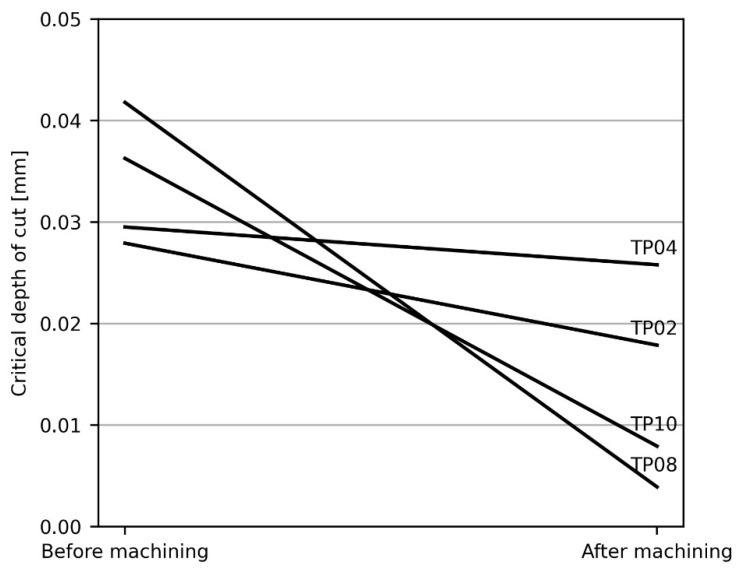
Critical axial depth of cut for the machining of each sample for different material removals.

The SLDs of the samples were calculated before, during, and after the pocketing tests at different axial depths of cut (Figure 5). SLDs show the frontier line under which axial depth of cut and spindle speed values lead to a chatter-free milling. As expected, the maximum chatter-free depth of cut is lower when machining has finalized, because the rigidity of the part is lower. Also, bigger material removals lead to lower maximum depths, as the decrease of the lobes is more pronounced for the cases of machining taking place at *a_p_* = 1 mm and *a_p_* = 0.8 mm. This phenomenon is more obvious if only the critical depth of cut is analyzed, namely, the lower maximum axial depth of cut that guarantees chatter-free milling irrespectively of spindle speed (Figure 6).

An important initial conclusion that can be derived from these SLDs is the difficulty to globally avoid chatter when a milling operation is performed in this kind of sample as they were set up. Even the machining conditions that according to the SLD initially avoid chatter (*a_p_* = 0.3 mm; 17,500 rpm) disappear because of material removal, which means that chatter would only be avoided at the beginning of the machining. In addition, the productivity would be low. Aiming to overcome these problems, a deeper analysis has been performed, studying separately each cutting pass of the machining path.

### 3.2. Vibration FFT Analysis

**Figure 7:**
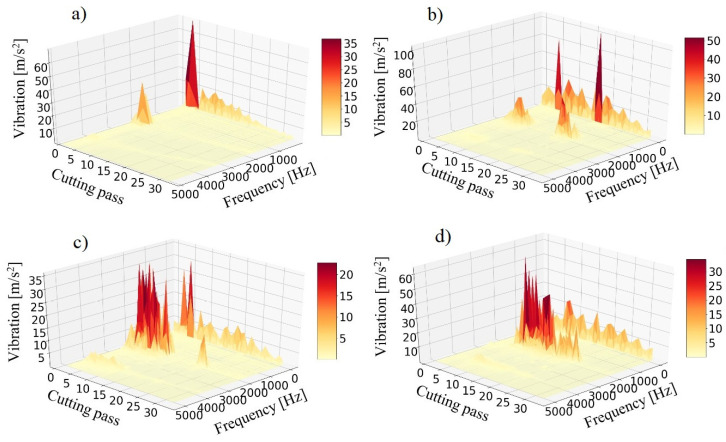
FFT of vibration signal of samples during machining. (**a**) TP10. (**b**) TP08. (**c**) TP04. (**d**) TP02.

The Fast Fourier Transform (FFT) of the vibration signal is a useful and representative indicator of the dynamic behavior of the system, which can be employed for determining the appearance of chatter vibration. In the current study, an FFT of the vibration of the samples, measured by the accelerometer placed below them during machining, has been obtained per cutting pass, namely, 33 FFT per sample. They are shown in three-dimensional plots in Figure 7. In these plots, the cutting passes where chatter takes place can be observed, as well as the frequency at which chatter occurs. Also, the vibration content of low frequencies is remarkable.

Three main conclusions can be obtained from these plots. Firstly, it is noticeable that there are two zones where chatter can be initialized. The first zone is around cutting passes 3–5, and the second zone is around cutting pass 21. For TP10, chatter only appears in the first zone. For TP08 and TP04, chatter appears in both zones, but disappears at the middle. For TP02, chatter appears almost continuously between these two zones.

The existence of these two zones is due to the complex interrelation of different facts. On the one hand, at the beginning of the machining in the center of the sample, despite starting far from fixtures, the rigidity of the sample is still high, and chatter does not occur. On the other hand, the presence of modal nodes, where rigidity is higher, and antinodes, where it is lower, in addition to the progressive variation of modal parameters, leads to the alternative appearance and disappearance of chatter along the tool path [31].

Secondly, it is noticeable that chatter appearance is higher for lower material removals; namely, it affects more cutting zones. As Campa et al. [32] have stated, this phenomenon happens in the milling of thin floors with bull-nose end mills, and it is related to the fact that low depths of cut also involve low lead edge angles, that ease chatter appearance.

And thirdly, it has been confirmed that chatter appears for all cases, as stated in the SLD analysis, but it disappears as long as the tool is reaching the fixtures. From cutting pass 25 the milling is stable for any depth of cut. That is to say that milling should be avoided in the central area between fixtures, but that milling is feasible and stable outside this area. In this case, the central area is approximately a central square of 30 × 30 mm^2^.

**Figure 8:**
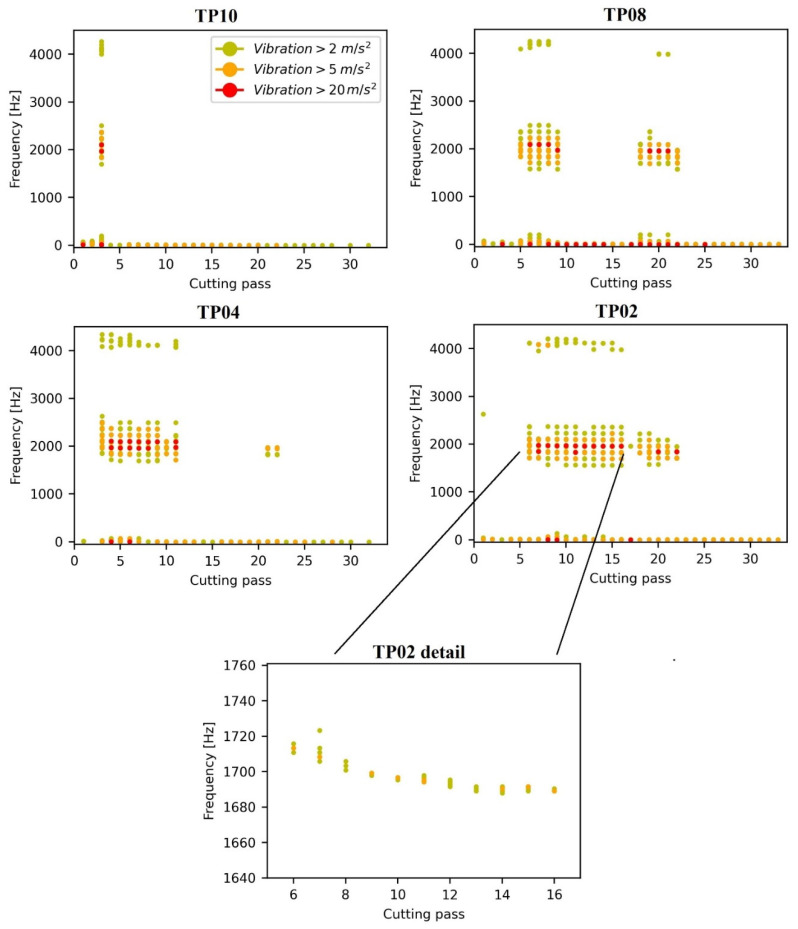
Frequencies with maximum vibration content per cutting pass, and TP02 detailed image.

The accurate chatter frequency can be more clearly seen in Figure 8, where only the highest vibration frequencies are shown. This chatter frequency is close to the first natural frequency of the samples, that according to the conducted FRF tests it is around 1730 Hz before machining. A second chatter frequency, close to the second natural frequency of 4000 Hz, is also excited. Both chatter frequencies are excited simultaneously, which discards the possibility of each zone being created by the vibration of different modes, as happened in thin wall studies [25,33].

Besides, in Figure 8 the harmonics of chatter frequency can be seen. These harmonics can be expressed by the following binomial:

(4) $$f_{chatter}+n\;\cdot \;f_{tooth}\;\left(n\;\in \;N\right)$$

It is remarkable that the first and second harmonics are higher than the chatter frequency. There are also low frequencies with high amplitude. These are the tooth passing frequency (133.33 Hz) and its harmonics, or they are related to the shape of the signal. In any case, they are higher for passes with chatter appearance, which indicates that chatter also affects the shape of the vibration signal.

Chatter frequencies decrease due to material removal, as natural frequency does, although this decrease is very slight and it is only appreciable for the highest material removals. The detailed image in Figure 8 illustrates this effect, as it shows the variation of the chatter frequency of TP02 during machining.

### 3.3. Roughness Analysis

**Figure 9:**
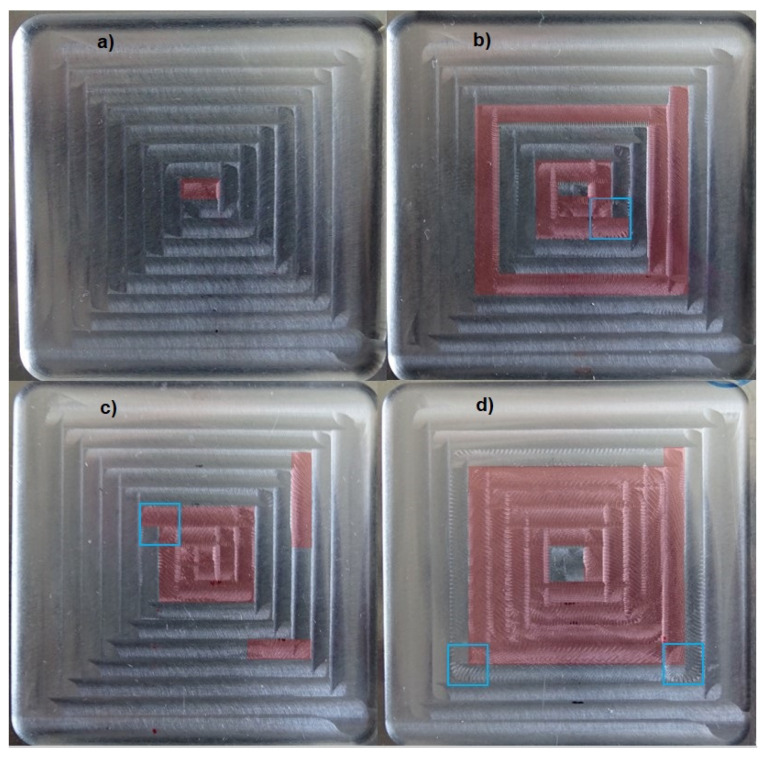
Samples after milling. Chatter marks in red, ploughing marks in blue square. (**a**) TP10. (**b**) TP08. (**c**) TP04. (**d**) TP02.

The final thin floors after milling are shown in Figure 9. In the four cases the first cutting pass does not exhibit chatter, but in the figure, due to the radial immersion of the successive passes, cutting pass 1 cannot be seen. In addition to chatter, there are ploughing marks at some changes of cutting direction.

**Figure 10:**
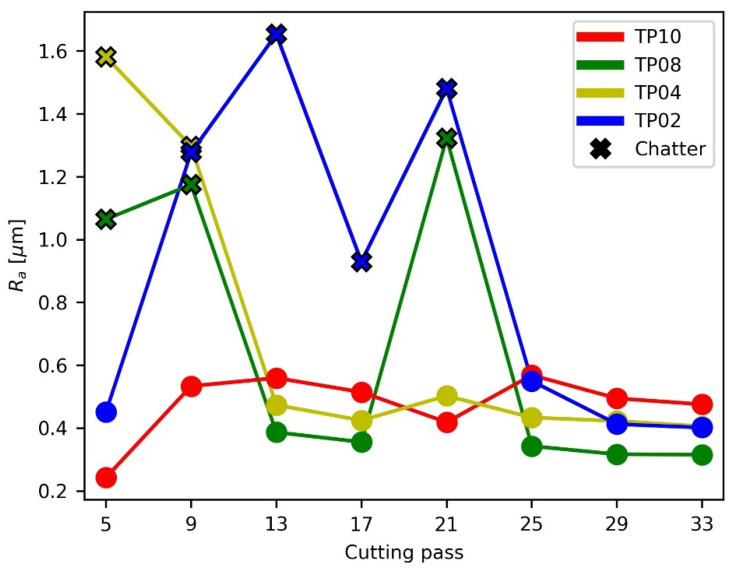
Average roughness per cutting pass.

The average roughness (*R_a_*) analysis (Figure 10) shows two types of passes clearly delimited. In the passes where chatter happens, the average roughness is higher than 0.9 μm, whereas in passes without chatter this value is lower than 0.6 μm. It must be noted that these values correspond to the middle of each cutting pass, where roughness has been measured.

Additionally, the roughness does not perceptibly change during machining. At most, it decreases very softly. This fact shows that, in absence of chatter, the roughness is affected by the *a_p_* employed, but it is not affected by the change of modal parameters, and it is only very slightly affected by the tool position and the proximity to fixtures. So, it can be concluded that the change of modal parameters affects chatter appearance, as stated in the previous SLD analysis, but once stability is reached surface roughness is constant and depends primarily on the machining parameters. Del Sol et al. [13] consider the idea that this happens due to the influence that machining parameters have on the cutting forces. In any case, given the current experimental setup, this achieved final surface quality is under 0.6 μm *R_a_* and thus it meets the industrial tolerances that are typically imposed on thin floors [13,24,34].

These results have also been compared to the available bibliography. Del Sol et al. [16] analyzed very similar kind of samples milled with the same mill, a feed rate of 0.08 mm/tooth and an axial depth of cut of 0.4 mm, although directly threaded to a plating sheet. Consequently, chatter does not appear in them. They exhibit an average roughness value of 0.25 μm measured in the direction of the milling. This value is lower than the average roughness value measured in the present case TP04 with a feed rate of 0.1 mm/tooth, which is around 0.4 μm. In a following experiment of Del Sol et al. [13], also with the same samples directly threaded to a plating sheet, average roughness varies between 0.2 μm and 0.4 μm. However, in this case roughness was not measured only in the direction of the milling, but also in the normal direction.

Rubio-Mateos et al. [14] studied the same type of samples milled with the same mill, clamped to a rubber-based vacuum fixture. Due to this fact, they do not exhibit chatter, and their average roughness value is between 0.4 and 0.6 μm for the same cutting conditions.

In another case, Campa et al. [32] milled various blocks of aluminum without back support from 30 mm thickness to 1 mm, employing a bull-nose end mill of 16 mm diameter, a feed of 0.05 mm/tooth, and depths of cut higher than 5 mm, as well as a parallel strategy (instead of helicoidal). The final average roughness was between 0.3 mm and 1.4 mm in absence of chatter. These results are consistent with the ones obtained in the present study, although the milling conditions were significantly different. Campa et al. [11] also conducted another similar experiment, where chatter marks were evident in some sections of the final part, as well as ploughing marks, a phenomenon in which the tool engages and penetrates on the part and that is related to the lack of stiffness. Arnaud et al. [25] consider ploughing as a type of process damping, in which the clearance face of the mill contacts the sample and thus leads to a more stable machining, a fact that can be confirmed in the present cases TP08 and TP04, where ploughing happens just before chatter disappearance.

In the present study, in addition to the lack of stiffness and to damping, it can be noticed in Figure 9 that ploughing is also related to the change of cutting direction. Milling strategies without sudden changes in the cutting direction, as circular ones, should be considered as they may avoid ploughing.

**Figure 11:**
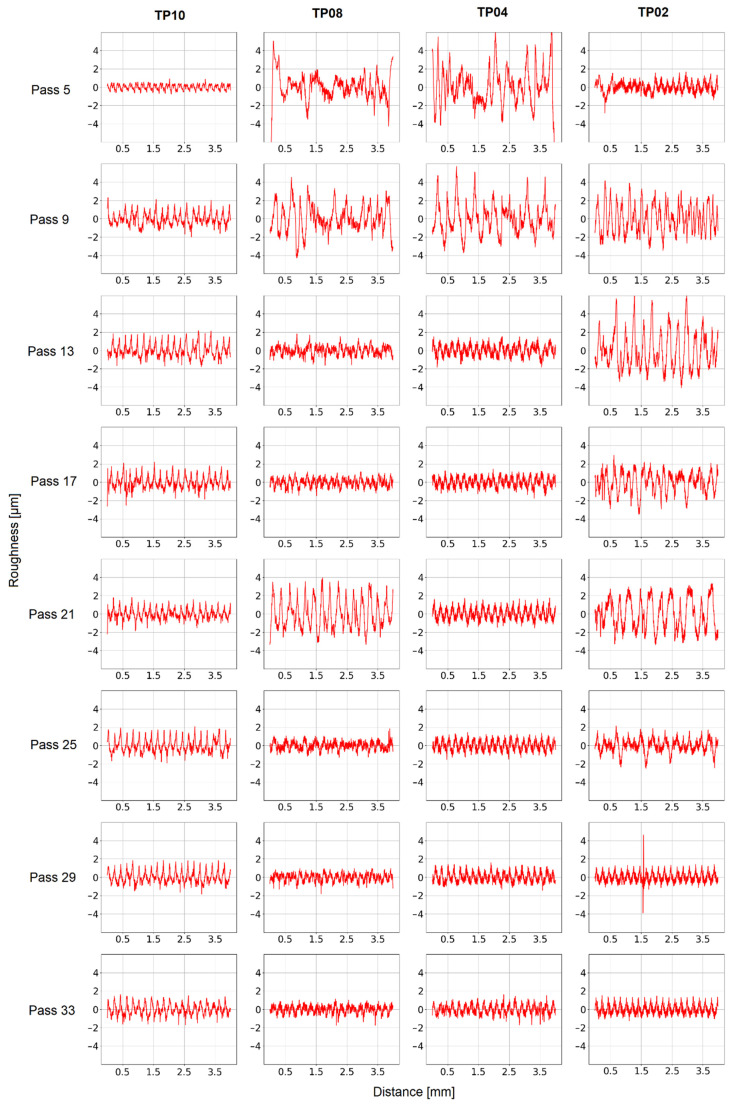
Roughness profile.

**Figure 12:**
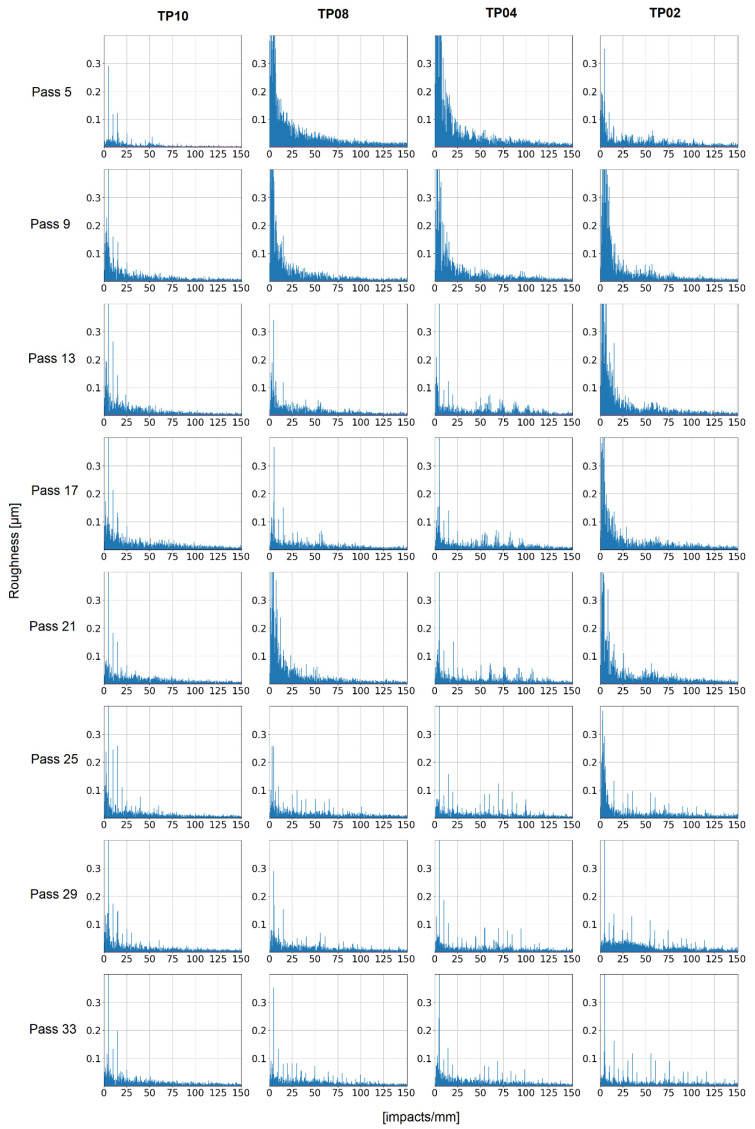
Roughness profile FFT.

The FFT analysis of the roughness profile of several cutting passes (Figure 11 and Figure 12) leads to two conclusions. On the one hand, the roughness caused by passes without chatter is mainly dominated by the tooth passing frequency (133.33 Hz, namely, 10 impacts/mm), the tool spin frequency (66.67 Hz, namely, 5 impacts/mm) and their harmonics. This phenomenon indicates that some tool runout is present during milling. These results also show that in the absence of chatter, surface roughness depends on the machining conditions. On the other hand, the roughness caused by passes with chatter appearance is higher and more chaotic. It does not appear at chatter frequencies (128 impacts/mm), but at low frequencies, even lower than the tooth passing frequency. It may be related to the previously described influence on the shape of the vibration signal caused by chatter.

**Figure 13:**
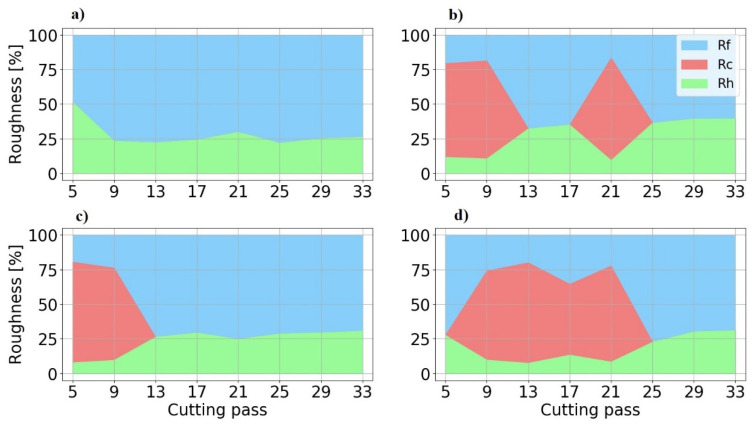
Average roughness components per cutting pass. (**a**) TP10. (**b**) TP08. (**c**) TP04. (**d**) TP02.

Surface roughness is the result of the combination of various factors acting simultaneously, as chatter and deflection, being difficult to discern between them, as López de Lacalle et al. [35] have pointed out. With the purpose of determining the origins of surface roughness, the roughness model proposed has been applied and its results are shown in Figure 13. In the case of chatter appearance, it causes up to 70% of roughness. In absence of chatter, the floor theoretical component of roughness (*R_h_*) is the smallest one and it varies from 36% in the lowest roughness case (TP08) to 25% in the highest one (TP10). In these cases, the displacement of fixtures and relative movement of thin plates (*R_f_*) cause at least 65% of *R_a_*.

**Figure 14:**
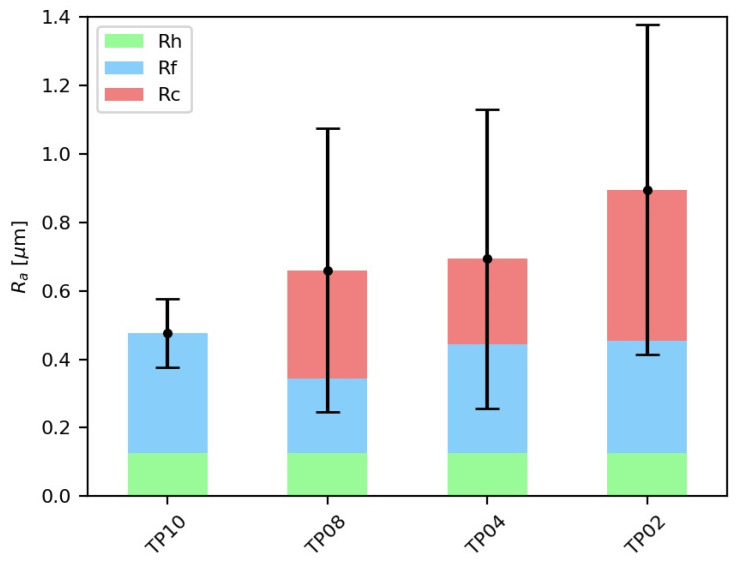
Components of the mean *R_a_* for each thin plate.

Figure 14 shows the mean and standard deviation values of *R_a_* for each thin plate, as well as their components. Low axial depths of cut lead to a high and variable roughness, whereas higher ones achieve smaller *R_a_* values. As previously stated while analyzing SLDs, this phenomenon happens because high axial depths of cut also entail high lead edge angles in bull-nose end mills, which make chatter appearance more difficult. Regarding *R_h_*, it is inherent to the employed mill and machining conditions, so it is constant in all thin plates, because the applied feed per tooth and mill are the same. The remaining roughness is mainly caused by forced vibrations and the axial displacement of the tool (*R_f_*); namely, it is attributable to the setup employed and the milled samples.

## 4. Conclusions

Chatter vibration and surface roughness resulting from the pocket milling of aluminum alloy thin plates without back support are studied. This situation of low rigidity is common between vacuum cups in flexible fixtures that support thin floors. Aiming to determine the conditions under which a milling in a situation of such a low stiffness could be stable and feasible, vibrations during machining and final surface roughness are measured and analyzed. SLDs are calculated, as well as the FFT of the vibration and the FFT of the surface roughness.

Main results and practical recommendations can be summarized as follows:

- Given the current experimental setup, chatter appearance in thin floors is dominated by the excitation of the first mode of vibration and, at the center of the sample, it is virtually unavoidable, irrespective of the axial depth of cut and spindle speed employed. This fact can be analytically deduced from stability lobes calculation, and it is empirically confirmed in the milling experiments. However, chatter can be avoided by selecting a proper machining zone. Actually, chatter is not a continuous phenomenon, but it appears and disappears along the cutting path due to the interaction of several factors. When an outward helicoidal milling strategy is performed, these factors are as follows: the removal of material, which reduces the rigidity of the samples, the approximation of the tool to the fixtures, which increases it, and the reaching of the tool to modal nodes and antinodes. As a consequence, in the current experimental setup chatter does not appear from cutting pass 25. It means that the proximity of the tool to the fixtures should be considered and incorporated to further chatter prediction models. Also, it means that flexible fixtures could be used for milling thin floors, providing that milling is not conducted in the central area between vacuum cups.
- Chatter is the main cause of poor surface quality for the studied cases, as it causes roughness of at least 0.9 μm in the current setup. In absence of chatter, irrespective of the depth of cut employed, roughness is always below 0.6 μm, so it would comply with industry quality requirements. As roughness caused by the displacement of fixtures and relative movement of thin plates is more than 60% of the global roughness, there is still potential for roughness reduction regarding clamping conditions. In those cases, roughness is dominated by the tooth passing frequency, so machining conditions can be employed to handle it.
- Given the current experimental setup, higher axial depths of cut (even 50% of the thickness of the sample) lead to a more stable machining than lower ones. It happens because higher depths of cut also entail higher lead edge angles, which avoid chatter. In addition to a more stable machining, higher depths of cut also lead to a more productive machining and to a surface roughness that complies with industry quality requirements.
- Process damping stabilizes the milling. However, it also entails ploughing effects, which are very harmful to surface quality. It is shown that the ploughing effect is a case of process damping also related to the change of cutting direction. Consequently, milling strategies without sudden changes in cutting direction should be considered as they may avoid ploughing.
- The methodology followed in the present study can be extended and applied to other aeronautic alloys, considering that they could have different surface quality requirements.

## Figures and Tables

**Table 1:** Conducted tasks.

Before Machining	During Machining	After Machining
Samples FRF measurement	Samples FRF measurement	SLD calculation
Tool FRF measurement	Vibration monitoring	Vibration FFT calculation
		Roughness measurement
		Roughness FFT calculation

**Table 2:** Thin plates according to the depth of cut.

Thin Plate Name	Axial Depth of Cut Employed
TP10	1 mm
TP08	0.8 mm
TP04	0.4 mm
TP02	0.2 mm

## Data Availability

Data available on request from the corresponding author.
